# Energy-Constrained LOCC-Assisted Quantum Capacity of the Bosonic Dephasing Channel

**DOI:** 10.3390/e25071001

**Published:** 2023-06-29

**Authors:** Amir Arqand, Laleh Memarzadeh, Stefano Mancini

**Affiliations:** 1 Department of Physics, Sharif University of Technology, Tehran 11365-9161, Iran; 2 School of Science & Technology, University of Camerino, I-62032 Camerino, Italy

**Keywords:** quantum channel maps, quantum capacities, squashed entanglement

## Abstract

We study the LOCC-assisted quantum capacity of a bosonic dephasing channel with energy constraint on the input states. We start our analysis by focusing on the energy-constrained squashed entanglement of the channel, which is an upper bound for the energy-constrained LOCC-assisted quantum capacity. As computing energy-constrained squashed entanglement of the channel is challenging due to a double optimization (over the set of density matrices and the isometric extensions of a squashing channel), we first derive an upper bound for it, and then, we discuss how tight that bound is for the energy-constrained LOCC-assisted quantum capacity of the bosonic dephasing channel. In doling so, we prove that the optimal input state is diagonal in the Fock basis. Then, we analyze two explicit examples of squashing channels through which we derive explicit upper and lower bounds for the energy-constrained LOCC-assisted quantum capacity of the bosonic dephasing channel in terms of its quantum capacity with different noise parameters. As the difference between upper and lower bounds becomes smaller by increasing the dephasing parameter, the bounds become tighter.

## 1. Introduction

One of the essential steps for the implementation of quantum information protocols and the development of quantum technology is the establishment of reliable communication between two parties. This is a motivator to analyze the capacity of quantum channels, especially quantum capacity, which corresponds to the highest rate at which quantum information can be communicated over many independent uses of a noisy quantum channel from a sender to a receiver (it also equals the highest rate at which entanglement can be generated over the channel).

As continuous-variable systems are promising candidates for quantum communication, analyzing the capacity of channels defined over infinite-dimensional Hilbert spaces is of practical and theoretical importance. In this set of channels, the subset of Gaussian channels that maps Gaussian states to Gaussian states has been studied extensively [1,2,3,4,5,6]. However, there is a strong motivation to go beyond Gaussian channels to have better performance in tasks such as parameter estimation [7] and teleportation [8,9,10] or to bypass the limitations of Gaussian maps for entanglement distillation [11,12,13,14], error correction [15], and quantum repeaters [16].

In general, computing quantum capacity is challenging because of two necessities: first, the optimization of an entropic functional (coherent information) over the set of input density operators, and second, its regularization [17]. The situation becomes more complicated for non-Gaussian channels compared to Gaussian ones because one cannot limit the analysis to states of Gaussian form, characterized just by covariance matrix and displacement vector. That makes obtaining analytical or numerical results for non-Gaussian channels a daunting task. Despite such technical difficulties, recently, there has been increasing attention to non-Gaussian channels [18,19,20,21]. In particular, in [20], it has been shown that the quantum capacity of bosonic dephasing channel, as an example of a non-Gaussian channel, is achieved by a Gaussian mixture of Fock states. Moreover, the quantum capacity of a deformed bosonic dephasing channel was recently studied in [22].

In [20], we addressed and derived the quantum capacity of the bosonic dephasing channel. It was shown [23] that the quantum capacity and LOCC-assisted quantum capacity of the bosonic dephasing channel are equal, and we thus conclude that the capacity of the channel does not increase under the allowance of LOCC assistance. Here, we are interested in finding the energy-constrained LOCC-assisted quantum capacity of the bosonic dephasing channel. The bosonic dephasing channel describes a snapshot of a quantum Markov process, and the channel noise parameter is proportional to the time the bosonic system interacts with the environment in the weak-coupling limit [20,24]. Furthermore, dephasing is an unavoidable source of noise in photonic communications [25]. This happens, for instance, with uncertainty path length in optical fibers [26].

Energy-constrained LOCC-assisted quantum capacity of the bosonic dephasing channel is the maximum rate at which entanglement can reliably be established between the sender and receiver when local operations and classical communication (LOCC) between the sender and receiver are also allowed. Additionally, we consider energy constraint on the channel input. The importance of sharing entanglement is related to the key role of this correlation in the implementation of quantum protocols. This is not limited to theoretical investigations and is actually the cornerstone of developing quantum networks [27]. This motivates analyzing any factor that affects the rate of entanglement sharing, including LOCC between the sender and the receiver.

Although for practical reasons it is essential to know the LOCC-assisted quantum capacity of the channels [28], there is no compact formula in terms of entropic functionals to quantifying it. It was proven that (energy constraint) squashed entanglement of a channel is an upper bound for (energy constraint) LOCC-assisted quantum capacity [29] and secret-key agreement capacity [28]. However, computing (energy constraint) squashed entanglement is another challenge because it requires two optimizations, one over the set of input density operators and another over the set of isometric extensions of a squashing channel. Thus, even computing a bound for the (energy constraint) LOCC-assisted quantum capacity through such optimization results, in general, is extremely challenging, if not impossible. In order to facilitate performing the optimization for computing the channel energy-constrained squashed-entanglement, we shall use the channel symmetry to restrict the search over smaller sets of density operators and isometric extensions. We will analytically prove that for 50/50 beamsplitter squashing channel, there is an upper bound and a lower bound for LOCC-assisted quantum capacity of the bosonic dephasing channel with/without energy constraint, in terms of its quantum capacity with/without energy constraint. Numerically we shall compute these bounds for inputs subject to energy constraint, which will result in tight bounds. We shall also discuss the value of these bounds when there is no input energy constraint. We also study symmetric qubit squashing channels and, in this subset, numerically search for the optimal squashing channel.

The structure of this paper is as follows. In Section 2, we set our notation and provide an essential background on squashed entanglement, LOCC-assisted quantum capacity, and the degradability of quantum channels. Here, we also recall the bosonic dephasing channel. In Section 3, we introduce the structure of the optimal input state. Section 4 is devoted to two explicit examples for squashing channels for the bosonic dephasing channel: 50/50 beamsplitter and symmetric qubit channels. We summarize and discuss the results in Section 5.

## 2. Background and Notation

In this section, we set our notation and provide the background required to follow the discussions in the next sections.

### 2.1. Notation

In this subsection, we set our notation. Throughout the paper we shall mainly deal with four input (output) systems. “*S*” and “$S^{\prime }$” label, respectively, the input and the output main system. Similarly, “*E*” and “$E^{\prime }$” label, respectively, the input and the output environment. “*R*” labels the reference system that remains unaltered from input to output. Finally, “*F*” and “$F^{\prime }$” denote the input and the output environment for the squashing channel that we shall introduce later on. The associated Hilbert spaces will be denoted by $H_{X}$ and $H_{X^{\prime }}$, where *X* can be either R,S,E,F or combinations of them. By $N_{X\to X^{\prime }}$, we denote a completely positive trace preserving (CPTP) map or, for short, a quantum channel [30]:

(1) $$N_{X\to X^{\prime }}:T\left(H_{X}\right)\to T\left(H_{X^{\prime }}\right),$$

where $T(H_{X})$ stands for the set of trace-class operators on $H_{X}$. Furthermore, by $L(H_{X})$, we represent the set of linear operators on the Hilbert space $H_{X}$.

A unitary extension of channel $N_{X\to X^{\prime }}$, is a unitary operator $U:H_{X}⊗H_{Y}\to H_{X^{\prime }}⊗H_{Y^{\prime }}$ where $H_{X}⊗H_{Y}$ is isomorphic with $H_{X^{\prime }}⊗H_{Y^{\prime }}$, such that [30]:

(2) $$\begin{array}{cc} N_{X\to X^{\prime }}\left(\rho_{X}\right) & ={\mathrm{Tr}}_{Y^{\prime }}\left(U_{XY\to X^{\prime }Y^{\prime }}^{N}\left(\rho_{X}⊗|0\rangle {\langle 0|}_{Y}\right)\right)={\mathrm{Tr}}_{Y^{\prime }}\left(U\left(\rho_{X}⊗|0\rangle {\langle 0|}_{Y}\right)U^{†}\right) \end{array}$$

for all $\rho_{X}\in T\left(H_{X}\right)$, where

(3) $$U_{XY\to X^{\prime }Y^{\prime }}^{N}\left[•\right]:=U•U^{†}.$$

Similarly, an isometric extension of channel $N_{X\to X^{\prime }}$ is an isometry $V:H_{X}\to H_{X^{\prime }}⊗H_{Y^{\prime }}$, such that [30]:

(4) $$N_{X\to X^{\prime }}\left(\rho_{X}\right)={\mathrm{Tr}}_{Y^{\prime }}\left(V_{X\to X^{\prime }Y^{\prime }}^{N}\left(\rho_{X}\right)\right)={\mathrm{Tr}}_{Y^{\prime }}\left(V\rho_{X}V^{†}\right),$$

for every $\rho_{X}\in T\left(H_{X}\right)$, where

(5) $$V_{X\to X^{\prime }Y^{\prime }}^{N}\left[•\right]:=V•V^{†}.$$

Purification of density matrix $\rho_{X}$ is denoted by $|\phi_{XY}\rangle$, and the density operator corresponding to it is [30]

(6) $$\phi_{XY}:=|\phi_{XY}\rangle \langle \phi_{XY}|.$$

The von Neumann entropy of an arbitrary state ρ is [30]

(7) S(ρ):=−Tr(ρlogρ).

Throughout the paper, we use the logarithm to base two. We recall that the von Neumann entropy is invariant under unitary transformations of the argument and is also sub-additive and strongly sub-additive [31,32].

The conditional entropy of a bipartite quantum state $\rho_{XY}$ is defined as follows [30]:

(8) $$S{\left(X|Y\right)}_{\rho_{XY}}:=S\left(\rho_{XY}\right)-S\left(\rho_{Y}\right),$$

where $\rho_{Y}={\mathrm{Tr}}_{X}(\rho_{XY}$). The quantity (8), unlike its classical counterpart, can be negative [31]. An additional property that will be used hereafter is its concavity.

For a bipartite quantum state $\rho_{SS^{\prime }}\in T\left(H_{S}⊗H_{S^{\prime }}\right)$, the mutual information $I{\left(S;S^{\prime }\right)}_{\rho_{SS^{\prime }}}$ quantifies the correlation between subsystems with reduced density matrices $\rho_{S}={\mathrm{Tr}}_{S^{\prime }}\left(\rho_{SS^{\prime }}\right)$ and $\rho_{S^{\prime }}={\mathrm{Tr}}_{S}\left(\rho_{SS^{\prime }}\right)$. It is defined as [30]:

(9) $$I{\left(S;S^{\prime }\right)}_{\rho_{SS^{\prime }}}:=S\left(\rho_{S}\right)+S\left(\rho_{S^{\prime }}\right)-S\left(\rho_{SS^{\prime }}\right).$$

This quantity, due to sub-additivity, is non-negative, like its classical counterpart [30,31].

Moreover, for a tri-partite quantum state $\rho_{SS^{\prime }R}\in T\left(H_{S}⊗H_{S^{\prime }}⊗H_{R}\right)$, conditional mutual information $I{\left(S;S^{\prime }|R\right)}_{\rho_{SS^{\prime }R}}$ quantifies the correlation between density matrices of subsystems $\rho_{S}={\mathrm{Tr}}_{RS^{\prime }}\left(\rho_{SS^{\prime }R}\right)$, and $\rho_{S^{\prime }}={\mathrm{Tr}}_{RS}\left(\rho_{SS^{\prime }R}\right)$, conditioned to $\rho_{R}={\mathrm{Tr}}_{SS^{\prime }}\left(\rho_{SS^{\prime }R}\right)$. This positive quantity is given by [30]

(10) $${I(S;S^{\prime }|R)}_{\rho_{SS^{\prime }R}}:=S{\left(S|R\right)}_{\rho_{SR}}+S{\left(S^{\prime }|R\right)}_{\rho_{S^{\prime }R}}-S{\left(SS^{\prime }|R\right)}_{\rho_{SS^{\prime }R}},$$

where the conditional entropy of a bipartite state is defined in Equation (8). The quantity (10) is non-negative because of the strong sub-additivity property [31,33].

### 2.2. Squashed Entanglement

In this subsection, we review the definition of quantities necessary for introducing the upper bound on the two-way LOCC-assisted quantum capacity of a channel. First, we recall the definition of squashed entanglement of a bipartite system. Then, we proceed with reviewing the definition of squashed entanglement of a channel and energy constraint squashed entanglement of a channel.

Squashed entanglement is an entanglement monotone for bipartite quantum states defined as follows [34]:

**Definition** **1.** *The squashed entanglement of a bipartite quantum state $\rho_{SS^{\prime }}\in T\left(H_{S}⊗H_{S^{\prime }}\right)$ is defined as*

(11) $$E_{sq}{\left(S;S^{\prime }\right)}_{\rho_{SS^{\prime }}}:=\frac{1}{2}\underset{\rho_{SS^{\prime }E^{\prime }}}{\inf}I{\left(S;S^{\prime }|E^{\prime }\right)}_{\rho_{SS^{\prime }E^{\prime }}},$$

*where the infimum is taken over all extensions of $\rho_{SS^{\prime }}$ that is over all quantum states $\rho_{SS^{\prime }E^{\prime }}$ such that $\rho_{SS^{\prime }}={\mathrm{Tr}}_{E^{\prime }}\left(\rho_{SS^{\prime }E^{\prime }}\right)$.*

Using the concept of bipartite state squashed entanglement, the squashed entanglement of a channel was introduced in [28]. It represents the maximum squashed entanglement that can be generated by the channel.

**Definition** **2.** *The squashed entanglement of a channel $N_{S\to S^{\prime }}$, is given by:*

(12) $${\tilde{E}}_{sq}\left(N_{S\to S^{\prime }}\right)=\underset{\rho_{S}}{\sup}E_{sq}\left(\rho_{S},N_{S\to S^{\prime }}\right),$$

*where the supremum is over all input density operators, $\rho_{S}\in T\left(H_{S}\right)$, and*

(13) $$\begin{array}{cc} E_{sq} & \left(\rho_{S},N_{S\to S^{\prime }}\right):=\frac{1}{2}\underset{V_{E\to E^{\prime }F^{\prime }}^{N_{E\to E^{\prime }}^{sq}}}{\inf}\left(S{\left(S^{\prime }|E^{\prime }\right)}_{\sigma_{S^{\prime }E^{\prime }}}+S{\left(S^{\prime }|F^{\prime }\right)}_{\sigma_{S^{\prime }F^{\prime }}}\right). \end{array}$$

**Figure 1:**
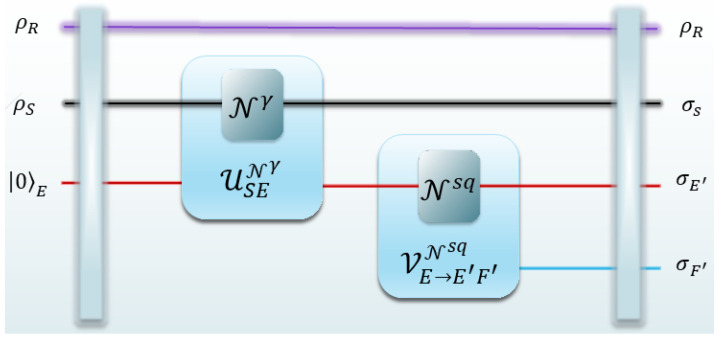
Schematic representation of the bosonic dephasing channel, squashing channel, and their isometric extensions.

*Here, the infimum is taken over all isometric extensions of the squashing channel (see (Figure 1)) and $\sigma_{S^{\prime }E^{\prime }}$ and $\sigma_{S^{\prime }F^{\prime }}$ are, respectively, obtained by partial trace over degrees of freedom in $H_{F^{\prime }}$ and $H_{E^{\prime }}$ of the state*

(14) $$\sigma_{S^{\prime }E^{\prime }F^{\prime }}:=\left(V_{E\to E^{\prime }F^{\prime }}^{N_{E\to E^{\prime }}^{sq}}\circ V_{S\to S^{\prime }E}^{N_{S\to S^{\prime }}}\right)\left(\rho_{S}\right),$$

*where $V_{S\to S^{\prime }E}^{N_{S\to S^{\prime }}}$ and $V_{E\to E^{\prime }F^{\prime }}^{N_{E\to E^{\prime }}^{sq}}$ are, respectively, the conjugation of isometric extension of the channel $N_{S\to S^{\prime }}$ and $N_{E\to E^{\prime }}^{sq}$ (see Equation (5)). The superscript sq in $N_{E\to E^{\prime }}^{sq}$ labels the squashing channel.*

If there exists a channel for which the infimum in Equation (13) is achieved, we call it the optimal squashing channel.

The definition of the squashed entanglement of a channel can be generalized to the case where there is a constraint on the energy of input states.

**Definition** **3.** *For channel $N_{S\to S^{\prime }}$ with energy constraint at the input, that is $\mathrm{Tr}(\rho_{S}G)\leq N$ where $\rho_{S}$ represents an arbitrary input state, G is the energy observable (the Hamiltonian of the system), and N∈[0,∞), the energy-constrained squashed entanglement of the channel is given by*

(15) $${\tilde{E}}_{sq}\left(N,G,N\right)=\underset{\rho_{S}:\mathrm{Tr}\left(\rho_{S}G\right)\leq N}{\sup}E_{sq}\left(\rho_{S},N_{S\to S^{\prime }}\right),$$

*where $E_{sq}\left(\rho_{S},N_{S\to S^{\prime }}\right)$ is defined in Equation (13).*

### 2.3. Two-Way LOCC-Assisted Quantum Capacity

In this subsection, we bring to light the definition of the two-way LOCC-assisted quantum capacity and its energy-constrained form [28,29]. Then, we recall its upper bound in terms of the squashed entanglement of a channel.

The performance of quantum channels for reliable quantum communication is quantified by quantum capacity when there are no extra resources, such as shared entanglement or classical communication between the sender and receiver. By allowing further resources, we expect higher rates of information transmission through the channel. When LOCC is allowed interactively between the sender and receiver, the capability of the channel for quantum communication is quantified by its two-way LOCC-assisted quantum capacity, which is defined as follows:

**Definition** **4.** *The two-way LOCC-assisted quantum capacity $Q_{S↔S^{\prime }}^{LOCC}\left(N_{S\to S^{\prime }}\right)$ of quantum channel $N_{S\to S^{\prime }}$ is the highest achievable rate of faithful qubit transmission (through infinitely many uses) of the channel with the assistance of unlimited two-way classical communication [35,36].*

The above definition is generalized for the situations where there is an upper bound on the average input energy:

**Definition** **5.** *The energy-constrained two-way LOCC-assisted quantum capacity $Q_{S↔S^{\prime }}^{LOCC}\left(N_{S\to S^{\prime }},G,N\right)$ of a quantum channel $N_{S\to S^{\prime }}$ is the two-way LOCC-assisted quantum capacity of Definition 4, with the constraint that the average input energy per channel use (determined by the observable G) is not larger than N.*

Note that we could have considered a uniform energy constraint at the input (constraining the energy of each input) instead of considering the average input energy constraint (see e.g., [29]). However, the two-way LOCC-assisted quantum capacity with uniform energy constraint is upper bounded by the two-way LOCC-assisted quantum capacity with average energy constraint on the input. To see this, note that the average input energy constraint on a state $\rho_{S_{1}\ldots S_{K}}$ over *K* channel uses can be written as

(16) $$\frac{1}{K}\sum_{i=1}^{K}\mathrm{Tr}\left(\rho_{S_{i}}G\right)\leq N,$$

where $\rho_{S_{i}}$ is the reduced state of $\rho_{S_{1}\ldots S_{K}}$ to the *i*-th input register and *G* is the Hamiltonian of each single input system. In contrast, the uniform input energy constraint reads

(17) $$\mathrm{Tr}\left(\rho_{S_{i}}G\right)\leq N,\;\;\forall i\in \left\{1,\ldots ,K\right\}.$$

It is easy to see that (17) is more demanding than (16) (see also [29]). Thus, deriving an upper bound on the former results in upper bounding the latter as well.

Despite the importance of two-way LOCC-assisted quantum capacity, there is no explicit compact expression to compute this capacity for a given channel. However, according to [29], an upper bound on $Q_{S↔S^{\prime }}^{LOCC}\left(N_{S\to S^{\prime }},G,N\right)$ is given by squashed entanglement of the channel:

(18) $$Q_{S↔S^{\prime }}^{LOCC}\left(N_{S\to S^{\prime }},G,N\right)\leq {\tilde{E}}_{sq}\left(N_{S\to S^{\prime }},G,N\right),$$

where the right hand is given in Equation (15).

### 2.4. Symmetric Channels

Here, we recall the notion of symmetric channels [37,38]. For defining symmetric channels, first the complementary channel needs to be introduced. For a channel

(19) $$\begin{array}{cc} \; & N_{X\to X^{\prime }}:•\mapsto {\mathrm{Tr}}_{Y}\left(U_{XY\to X^{\prime }Y^{\prime }}^{N}\left(•⊗|0\rangle \langle 0|\right)\right), \end{array}$$

with $U_{XY\to X^{\prime }Y^{\prime }}^{N}$ defined in Equation (3), the complementary channel $N_{X\to Y^{\prime }}^{c}$ is given by

(20) $$\begin{array}{cc} \; & N_{X\to Y^{\prime }}^{c}:•\mapsto {\mathrm{Tr}}_{X}\left(U_{XY\to X^{\prime }Y^{\prime }}^{N}\left(•⊗|0\rangle \langle 0|\right)\right), \end{array}$$

Setting the definition of complementary channel, symmetric channels are those channels for which

(21) $$N_{X\to Y^{\prime }}^{c}=N_{X\to X^{\prime }}.$$

Indeed, for symmetric channels $T(H_{X^{\prime }})$ and $T(H_{Y^{\prime }})$ are isomorphic.

### 2.5. Quantum Dephasing Channel

The continuous-variable bosonic dephasing channel $N_{S\to S^{\prime }}^{\gamma }$ can successfully model decoherence in many different setups [39]. As the input space $H_{S}$ and output space $H_{S^{\prime }}$ are isomorphic, from now on, we denote the bosonic dephasing channel with $N_{S\to S}^{\gamma }$ where γ∈[0,+∞) is related to the dephasing rate. Bosonic dephasing channels are described through the following operator–sum representation [20].

(22) $$N_{S\to S}^{\gamma }\left(\rho \right)=\sum_{j=0}^{\infty }K_{j}\rho K_{j}^{†},$$

where the Kraus operators are given by

(23) $$K_{j}=e^{-\frac{1}{2}\gamma {\left(a^{†}a\right)}^{2}}\frac{{\left(-i\sqrt{\gamma }a^{†}a\right)}^{j}}{\sqrt{j!}},$$

with $a^{†},a$ being bosonic creation and annihilation operators on $H_{S}$, and γ∈[0,+∞) is related to the dephasing rate. To see how γ is related to the dephasing rate, let us look at another equivalent representation of the channel [20]:

(24) $$N_{S\to S}^{\gamma }\left(\rho \right)=\int_{-\infty }^{+\infty }e^{-ia^{†}a\phi }\rho e^{ia^{†}a\phi }p_{\gamma }\left(\phi \right)d\phi ,$$

with

(25) $$p_{\gamma }\left(\phi \right)=\sqrt{\frac{1}{2\pi \gamma }}e^{-\frac{\phi^{2}}{2\gamma }}.$$

As can be seen from (24), each $e^{-ia^{†}a\phi }$ term corresponds to a phase shift ϕ to the input state ρ. Thus, the channel is a probabilistic mixture of phase-shift operators to the input state with a probability distribution $p_{\gamma }\left(\phi \right)$ that depends on γ, (which is actually the variance of the probability dephasing distribution).

The channel can be dilated into a single-mode environment using the following unitary $U_{N_{S\to S}^{\gamma }}\in L\left(H_{S}⊗H_{E}\right)$

(26) $$\begin{array}{c} U_{N_{S\to S}^{\gamma }}=e^{-i\sqrt{\gamma }\left(a^{†}a\right)\left(b+b^{†}\right)}=e^{-i\sqrt{\gamma }\left(a^{†}a\right)b^{†}}e^{-i\sqrt{\gamma }\left(a^{†}a\right)b}e^{-\frac{1}{2}\gamma {\left(a^{†}a\right)}^{2}}, \end{array}$$

with $b^{†},b$ being bosonic creation and annihilation operators on $H_{E}$. The unitary (26) has the form of a controlled dephasing with the environment’s mode acting as a control.

It is not hard to see that the channel $N_{S\to S}^{\gamma }$ has a phase covariant property under the unitary operator, that is

(27) $$N_{S\to S}^{\gamma }\left(U_{\theta }\rho U_{\theta }^{†}\right)=U_{\theta }N_{S\to S}^{\gamma }\left(\rho \right)U_{\theta }^{†},$$

where unitary operator $U_{\theta }$ is given by

(28) $$U_{\theta }=e^{i(a^{†}a)\theta }\in L(H_{S}).$$

Moreover, given $\rho =\sum_{m,n}\rho_{m,n}\left|m\rangle \langle n\right|$, the output of the complementary channel can be written as

(29) $$\begin{array}{c} N_{S\to E}^{\gamma^{c}}\left(\rho \right)={\mathrm{Tr}}_{S}\left[U_{N_{S\to S}^{\gamma }}\left(\rho ⊗|0\rangle \langle 0|\right)U_{N_{S\to S}^{\gamma }}^{†}\right]=\sum_{n=0}^{\infty }\rho_{n,n}|-i\sqrt{\lambda }n\rangle \langle -i\sqrt{\lambda }n|, \end{array}$$

where $|-i\sqrt{\lambda }n\rangle \in H_{E}$ is a coherent state of amplitude $\sqrt{\lambda }n$ with phase −i and |0〉 is the vacuum state of the environment. By the above relation and using Equation (28), we can see that

(30) $$N_{S\to E}^{\gamma^{c}}\left(U_{\theta }\rho U_{\theta }^{†}\right)=N_{S\to E}^{\gamma^{c}}\left(\rho \right).$$

which means that the complementary channel of $N_{S\to E}^{\gamma }$ is invariant under the unitary (28).

## 3. Optimal Input State

In this section, we derive an upper bound for the squashed entanglement of the bosonic dephasing channel defined in Equation (22). In doing so, we prove that the optimal input state for which such an upper bound can be achieved is diagonal in the Fock basis. We use the structure of optimal input state to simplify the expression for squashed entanglement of the channel, which we will use in subsequent sections.

To analyze energy-constrained squashed entanglement (see Definition 3) for the bosonic dephasing channel as energy observable *G*, we use the operator $a^{†}a$ because for a bosonic mode, it corresponds (up to a constant) to the Hamiltonian.

**Proposition** **1.** *For a bosonic dephasing channel with parameter γ and energy observable $G=a^{†}a$, the supremum in Equation (15) is achieved by diagonal states in the Fock basis.*

**Proof** **of** **Proposition** **1.** Define $U_{SX}:\left(H_{S}⊗H_{X}\right)\to \left(H_{S}⊗H_{X}\right)$ to be

(31) $$U_{SX}=U_{\theta }⊗{\mathrm{id}}_{X},$$

where $U_{\theta }$ is as in Equation (28). Moreover, consider an arbitrary joint density operator $\sigma_{SX}\in D\left(H_{S}⊗H_{X}\right)$ and denote

(32) $$\sigma_{SX}^{\theta }:=U_{SX}\sigma_{SX}U_{SX}^{†}.$$

Due to the invariance property of von Neumann entropy under unitary transformations, we have

(33) $$\begin{array}{c} S{\left(S|E^{\prime }\right)}_{\sigma_{SE^{\prime }}}+S{\left(S|F^{\prime }\right)}_{\sigma_{SF^{\prime }}}=S{\left(S|E^{\prime }\right)}_{\sigma_{SE^{\prime }}^{\theta }}+S{\left(S|F^{\prime }\right)}_{\sigma_{SF^{\prime }}^{\theta }}, \end{array}$$

where $\sigma_{SE^{\prime }}^{\theta }$ and $\sigma_{SF^{\prime }}^{\theta }$ are defined in Equation (32) with the Hilbert space $H_{X}$ to be $H_{E^{\prime }}$ and $H_{F^{\prime }}$, respectively, and

(34) $$\begin{array}{cc} \; & \sigma_{SE^{\prime }}:=\left({\mathrm{id}}_{S}⊗N_{E\to E^{\prime }}^{sq}\right)\left(U_{N_{S\to S}^{\gamma }}\rho_{SE}U_{N_{S\to S}^{\gamma }}^{†}\right), \end{array}$$

(35) $$\begin{array}{cc} \; & \sigma_{SF^{\prime }}:=\left({\mathrm{id}}_{S}⊗N_{E\to F^{\prime }}^{sq^{c}}\right)\left(U_{N_{S\to S}^{\gamma }}\rho_{SE}U_{N_{S\to S}^{\gamma }}^{†}\right), \end{array}$$

with $\rho_{SE}\in T(H_{S}⊗H_{E})$ being an arbitrary system–environment initial state. On the other hand, the conditional entropy is concave, meaning that the following relation holds true:

(36) $$\begin{array}{c} \frac{1}{2\pi }\int_{0}^{2\pi }d\theta \left(S{\left(S|E^{\prime }\right)}_{\sigma_{SE^{\prime }}^{\theta }}+S{\left(S|F^{\prime }\right)}_{\sigma_{SF^{\prime }}^{\theta }}\right)\leq S{\left(S|E^{\prime }\right)}_{{\bar{\sigma }}_{SE^{\prime }}}+S{\left(S|F^{\prime }\right)}_{{\bar{\sigma }}_{SF^{\prime }}}, \end{array}$$

where

(37) $$\begin{array}{cc} \; & {\bar{\sigma }}_{SE^{\prime }}:=\left({\mathrm{id}}_{S}⊗N_{E\to E^{\prime }}^{sq}\right)\left(U_{N_{S\to S}^{\gamma }}\left(\frac{1}{2\pi }\int_{0}^{2\pi }d\theta \rho_{SE}^{\theta }\right)U_{N_{S\to S}^{\gamma }}^{†}\right), \end{array}$$

(38) $$\begin{array}{cc} \; & {\bar{\sigma }}_{SF^{\prime }}:=\left({\mathrm{id}}_{S}⊗N_{E\to F^{\prime }}^{sq^{c}}\right)\left(U_{N_{S\to S}^{\gamma }}\left(\frac{1}{2\pi }\int_{0}^{2\pi }d\theta \rho_{SE}^{\theta }\right)U_{N_{S\to S}^{\gamma }}^{†}\right), \end{array}$$

with $\rho_{SE}^{\theta }$ defined in the same way as in Equation (32). Considering Equations (26) and (31), with the aid of simple algebraic steps, it can be seen that the following commutation relation holds true:

(39) $$[U_{N_{S\to S}^{\gamma }},U_{SE}]=0.$$

This means that the unitary extension of the phase covariant bosonic dephasing channel is invariant under a local phase operator and is symmetric. Using this commutation relation, we can conclude that:

(40) $$\begin{array}{cc} \; & U_{SE^{\prime }}\left({\mathrm{id}}_{S}⊗N_{E\to E^{\prime }}^{sq}\right)U_{N_{S\to S}^{\gamma }}=\left({\mathrm{id}}_{S}⊗N_{E\to E^{\prime }}^{sq}\right)U_{N_{S\to S}^{\gamma }}U_{SE}, \end{array}$$

(41) $$\begin{array}{cc} \; & U_{SF^{\prime }}\left({\mathrm{id}}_{S}⊗N_{E\to F^{\prime }}^{sq^{c}}\right)U_{N_{S\to S}^{\gamma }}=\left({\mathrm{id}}_{S}⊗N_{E\to F^{\prime }}^{sq^{c}}\right)U_{N_{S\to S}^{\gamma }}U_{SE}. \end{array}$$

Then, by means of Equation (33), relation (36) becomes:

(42) $$S{\left(S|E^{\prime }\right)}_{\sigma_{SE^{\prime }}}+S{\left(S|F^{\prime }\right)}_{\sigma_{SF^{\prime }}}\leq S{\left(S|E^{\prime }\right)}_{{\bar{\sigma }}_{SE^{\prime }}}+S{\left(S|F^{\prime }\right)}_{{\bar{\sigma }}_{SF^{\prime }}}.$$

Now, consider the initial joint state of the system and the environment to be

(43) $$\rho_{SE}=\rho_{S}⊗{|0\rangle }_{E}\langle 0|.$$

By expanding $\rho_{S}$ in the Fock basis as $\rho_{S}=\sum_{n,m}\rho_{m,n}|n\rangle \langle m|$, the integrals in Equations (37) and (38) take the following form:

(44) $$\begin{array}{cc} \frac{1}{2\pi }\int_{0}^{2\pi }d\theta \rho_{SE}^{\theta } & =\frac{1}{2\pi }\int_{0}^{2\pi }d\theta U_{SE}\left(\rho_{S}⊗{|0\rangle }_{E}\langle 0|\right)U_{SE}^{†}\\ \; & =\frac{1}{2\pi }\sum_{m,n=0}^{\infty }\int_{0}^{2\pi }d\theta \rho_{n,m}e^{i(n-m)\theta }{|n\rangle }_{S}\langle m|⊗{|0\rangle }_{E}\langle 0|\\ \; & =\sum_{n=0}^{\infty }\rho_{n,n}{|n\rangle }_{S}\langle n|⊗{|0\rangle }_{E}\langle 0|. \end{array}$$

Therefore, by considering the above relation along with Equation (42), it can be seen that the optimal input state for squashed entanglement of the channel defined in Equation (12) is diagonal in the Fock basis.

For the case where we have energy constraint on the input state, the same arguments from Equation (31) to Equation (44) hold true. However, this time, the optimal input state in Equation (44) takes the form

(45) $$\rho_{SE}^{opt}={\sum_{n}}^{\prime }\rho_{n,n}{|n\rangle }_{S}\langle n|⊗{|0\rangle }_{E}\langle 0|,$$

where

(46) $${\sum_{n}}^{\prime }:=\sum_{\begin{array}{c} n=0\\ \mathrm{Tr}(\rho_{S}a^{†}a)\leq N \end{array}}^{\infty },$$

and we shall use this notation hereafter. □

The proof of Proposition 1 is based on two main properties. The first one is the concavity of conditional entropy. Similar arguments have been used to bound the squashed entanglement of other channels [29]. The second one is the direct usage of the symmetry property of the unitary extension of the phase covariant bosonic dephasing channel, without invoking the fact that the isometric extension of a group covariant channel has covariant properties [40].

Due to Proposition 1, the supremum in Equation (15) is replaced by a supremum over the set of diagonal states in the Fock basis satisfying the energy constraint, or in other words, over the probability distributions of Fock states satisfying the energy constraint:

(47) $${\tilde{E}}_{sq}\left(N_{S\to S}^{\gamma },a^{†}a,N\right)=\underset{p_{n}:\sum_{n}np_{n}\leq N}{\sup}E_{sq}\left(\rho_{S}^{opt},N_{S\to S}^{\gamma }\right),$$

where

(48) $$\rho_{S}^{opt}={\sum_{n}}^{\prime }p_{n}{|n\rangle }_{S}\langle n|,\;p_{n}:=\rho_{n,n},$$

is obtained by tracing over the environment degrees of freedom of Equation (45). Hence, for the optimal input state, the system–environment output state is given by

(49) $$\sigma_{SE^{\prime }}^{opt}={\sum_{n}}^{\prime }p_{n}{|n\rangle }_{S}\langle n|⊗{|-i\sqrt{\gamma }n\rangle }_{E}\langle -i\sqrt{\gamma }n|.$$

For subsequent developments, it is more convenient to re-express Equation (13) in terms of mutual information, namely:

(50) $$\begin{array}{cc} E_{sq}\left(\rho_{S},N_{S\to S^{\prime }}\right)\; & =\underset{V_{E\to E^{\prime }F^{\prime }}^{N_{E\to E^{\prime }}^{sq}}}{\inf}\frac{1}{2}\left(S{\left(S^{\prime }|E^{\prime }\right)}_{\sigma_{S{}^{\prime }E^{\prime }}}+S{\left(S{}^{\prime }|F^{\prime }\right)}_{\sigma_{S{}^{\prime }F^{\prime }}}\right)\\ \; & =\underset{V_{E\to E^{\prime }F^{\prime }}^{N_{E\to E^{\prime }}^{sq}}}{\inf}\frac{1}{2}\left(S\left(\sigma_{S{}^{\prime }E^{\prime }}\right)-S\left(\sigma_{E^{\prime }}\right)+S\left(\sigma_{S{}^{\prime }F^{\prime }}\right)-S\left(\sigma_{F^{\prime }}\right)\right)\\ \; & =S\left(\sigma_{S{}^{\prime }}\right)-\underset{V_{E\to E^{\prime }F^{\prime }}^{N_{E\to E^{\prime }}^{sq}}}{\sup}\frac{1}{2}\left(I{\left(S{}^{\prime };E^{\prime }\right)}_{\sigma_{S{}^{\prime }E^{\prime }}}+I{\left(S{}^{\prime };F^{\prime }\right)}_{\sigma_{S{}^{\prime }F^{\prime }}}\right), \end{array}$$

Therefore, for the bosonic dephasing channel with an optimal input state, we have:

(51) $$\begin{array}{c} E_{sq}\left(\rho_{S}^{opt},N_{S\to S}^{\gamma }\right)=S\left(\sigma_{S}^{opt}\right)-\underset{V_{E\to E^{\prime }F^{\prime }}^{N_{E\to E^{\prime }}^{sq}}}{\sup}\frac{1}{2}\left(I{\left(S;E^{\prime }\right)}_{\sigma_{SE^{\prime }}^{opt}}+I{\left(S;F^{\prime }\right)}_{\sigma_{SF^{\prime }}^{opt}}\right), \end{array}$$

where due to the invariance of an optimal input state under channel action, the output of the channel is given by $\sigma_{S}^{opt}=\rho_{S}^{opt}$, and

(52) $$\begin{array}{cc} \; & \sigma_{SE^{\prime }}^{opt}={\sum_{n}}^{\prime }p_{n}{|n\rangle }_{S}\langle n|⊗N_{E\to E^{\prime }}^{sq}\left(|-i\sqrt{\gamma }n\rangle \langle -i\sqrt{\gamma }n|\right), \end{array}$$

(53) $$\begin{array}{cc} \; & \sigma_{SF^{\prime }}^{opt}={\sum_{n}}^{\prime }p_{n}{|n\rangle }_{S}\langle n|⊗N_{E\to F^{\prime }}^{sq^{c}}\left(|-i\sqrt{\gamma }n\rangle \langle -i\sqrt{\gamma }n|\right), \end{array}$$

are classical quantum states.

In this section, we investigated the squashed entanglement of a bosonic dephasing channel, which is an upper bound for its energy-constrained two-way LOCC-assisted quantum capacity (Equation (18)) [29]. We showed that to compute this upper bound, two optimizations are required: one over the probability distribution of Fock states at the input (see Equation (47)) and the other over isometric extensions of the squashing channel (see Equation (51)).

## 4. Squashing Channel for Bosonic Dephasing Channel

Confining our search for squashing channels to the set of symmetric channels, Equation (51) turns into:

(54) $$\begin{array}{c} E_{sq}\left(\rho_{S}^{opt},N_{S\to S}^{\gamma }\right)\leq S\left(\sigma_{S}^{opt}\right)-\underset{V_{E\to E^{\prime }F^{\prime }}^{N_{E\to E^{\prime }}^{sq}}\in V_{sym}}{\sup}\frac{1}{2}\left(I{\left(S;E^{\prime }\right)}_{\sigma_{SE^{\prime }}^{opt}}+I{\left(S;F^{\prime }\right)}_{\sigma_{SF^{\prime }}^{opt}}\right) \end{array}$$

where $\sigma_{SE^{\prime }}^{opt}$ is defined in Equation (52), and $V_{sym}$ is the set of isometric dilations of symmetric channels. When the squashing channel belongs to the set of symmetric channels, $\sigma_{SE^{\prime }}^{opt}=\sigma_{SF^{\prime }}^{opt}$. Hence, we have

(55) $$\begin{array}{cc} E_{sq}\left(\rho_{S}^{opt},N_{S\to S}^{\gamma }\right)\; & \leq S\left(\sigma_{S}^{opt}\right)-\underset{V_{E\to E^{\prime }F^{\prime }}^{N_{E\to E^{\prime }}^{sq}}\in V_{sym}}{\sup}I{\left(S;E^{\prime }\right)}_{\sigma_{SE^{\prime }}^{opt}}\\ \; & =S\left(\sigma_{S}^{opt}\right)-\underset{N_{E\to E^{\prime }}^{sq}\in N_{sym}}{\sup}I{\left(S;E^{\prime }\right)}_{\sigma_{SE^{\prime }}^{opt}} \end{array}$$

where $N_{sym}$ is the set of symmetric channels. The last equality holds true because the mutual information $I{\left(S;E^{\prime }\right)}_{\sigma_{SE^{\prime }}^{opt}}$ only depends on the squashing channel, not on its isometric extension.

In the next coming subsections, we consider two specific cases. In the first one, we consider a 50/50 beamsplitter for the symmetric squashing channel, and in the second one, we restrict the search for the optimal squashing channel to the set of symmetric qubit channels.

### 4.1. 50/50 Beamsplitter Squashing Channel

In this subsection, we consider a 50/50 beamsplitter as the squashing channel. Among one-mode Gaussian symmetric channels, the most well-known is the 50/50 beamsplitter. Furthermore, this choice is in line with the results in [28], where it is shown that in the set of pure-loss channels, the 50/50 beamsplitter is the optimal squashing channel.

A beamsplitter has two inputs, one playing the role of the environment and the other of the input to the channel. When the environment mode is kept in a vacuum state, the beamsplitter performs as a Gaussian channel and is described by the map [41]:

(56) $$N_{\eta }^{BS}\left(\rho \right)=\sum_{k=0}^{\infty }B_{k}\left(\eta \right)\rho B_{k}^{†}\left(\eta \right),$$

where η∈(0,1) is the transmissivity of the beamsplitter, and $B_{k}\left(\eta \right)$s are the Kraus operators taking the following explicit form in the Fock basis:

(57) Bk(η)=∑m=0∞m+kk(1−η2)k2ηm|m〉〈m+k|.

The beamsplitter transforms a single-mode coherent input state |β〉 into a single-mode coherent output state |ηβ〉, with a smaller amplitude [41]:

(58) $$N_{\eta }^{BS}\left(|\beta \rangle \langle \beta |\right)=|\eta \beta \rangle \langle \eta \beta |.$$

In this representation, a 50/50 beamsplitter corresponds to $\eta =\frac{1}{\sqrt{2}}$. Therefore, according to Equations (47) and (51), an upper bound on the squashed entanglement of the bosonic dephasing channel can be obtained by the following relation:

(59) $${\tilde{E}}_{sq}\left(N_{S\to S}^{\gamma },a^{†}a,N\right)\leq \underset{p_{n}}{\sup}\left(S\left(\sigma_{S}^{opt}\right)-I{\left(S;E^{\prime }\right)}_{\sigma_{SE^{\prime }}^{opt}}\right),$$

where $\sigma_{S}^{opt}$ and $\sigma_{E^{\prime }}^{opt}$ are obtained by partially tracing the following density operator with respect to *S* and $E^{\prime }$

(60) $$\begin{array}{cc} \sigma_{SE^{\prime }}^{opt} & ={\sum_{n}}^{\prime }p_{n}{|n\rangle }_{S}\langle n|⊗N_{\frac{1}{\sqrt{2}}}^{BS}\left({|-i\sqrt{\gamma }n\rangle }_{E^{\prime }}\langle -i\sqrt{\gamma }n|\right)\\ & ={\sum_{n}}^{\prime }p_{n}{|n\rangle }_{S}\langle n|⊗{|-\frac{i}{\sqrt{2}}\sqrt{\gamma }n\rangle }_{E^{\prime }}\langle -\frac{i}{\sqrt{2}}\sqrt{\gamma }n|. \end{array}$$

As $\sigma_{SE^{\prime }}^{opt}$ is a separable state,

(61) $$\begin{array}{c} S\left(\sigma_{S}^{opt}\right)-I{\left(S;E^{\prime }\right)}_{\sigma_{SE^{\prime }}^{opt}}=S\left(\sigma_{SE^{\prime }}^{opt}\right)-S\left(\sigma_{E^{\prime }}^{opt}\right)=S\left(\sigma_{S}^{opt}\right)-S\left(\sigma_{E^{\prime }}^{opt}\right). \end{array}$$

Therefore, Equation (59) is simplified to

(62) $$\begin{array}{cc} {\tilde{E}}_{sq}\left(N_{S\to S}^{\gamma },a^{†}a,N\right) & \leq \underset{p_{n}}{\sup}\left(S\left(\sigma_{S}^{opt}\right)-S\left(\sigma_{E^{\prime }}^{opt}\right)\right). \end{array}$$

From Equations (60) and (62), it is concluded that:

(63) $$\begin{array}{cc} {\tilde{E}}_{sq}\left(N_{S\to S}^{\gamma },a^{†}a,N\right) & \leq \underset{p_{n}}{\sup}\left(S\left({\sum_{n}}^{\prime }p_{n}|n\rangle \langle n|\right)-S\left({\sum_{n}}^{\prime }p_{n}|\frac{n}{i}\sqrt{\frac{\gamma }{2}}\rangle \langle \frac{n}{i}\sqrt{\frac{\gamma }{2}}n|\right)\right) \end{array}$$

(64) $$\begin{array}{cc} & \;\;\;\;\;\;\;=\underset{p_{n}}{\sup}\left(S\left({\sum_{n}}^{\prime }p_{n}|n\rangle \langle n|\right)-S\left({\sum_{n}}^{\prime }p_{n}|-\sqrt{\frac{\gamma }{2}}n\rangle \langle -\sqrt{\frac{\gamma }{2}}n|\right)\right). \end{array}$$

Equality (64) is due to the invariance property of von Neumann entropy under the unitary conjugation that transforms coherent state |iα〉 to |α〉, ∀α∈C. In [20], it is shown that the right-hand side of Equation (64) is the (unassisted) quantum capacity *Q* of a bosonic dephasing channel with a dephasing parameter $\frac{\gamma }{2}$ and the energy constraint *N*. Thus,

(65) $$\begin{array}{c} Q_{S↔S}^{LOCC}\left(N_{S\to S}^{\gamma },a^{†}a,N\right)\leq {\tilde{E}}_{sq}\left(N_{S\to S}^{\gamma },a^{†}a,N\right)\leq Q\left(N_{S\to S}^{\frac{\gamma }{2}},a^{†}a,N\right). \end{array}$$

Thus far, we have derived an upper bound for the energy-constrained squashed entanglement of the channel, which in turn, is an upper bound for the energy-constrained two-way LOCC-assisted capacity. Next we derive a lower bound for the energy-constrained two-way LOCC-assisted capacity. In [42], a lower bound on the two-way LOCC-assisted quantum capacity was introduced with the name of reverse coherent information [43,44,45]. The reverse coherent information of a channel $N_{S\to S^{\prime }}$ is defined as

(66) $$I_{R}\left(N_{S\to S^{\prime }}\right):=\underset{\rho_{S}}{\sup}\left(S\left(\rho_{S}\right)-S\left(N_{S\to E}^{c}\left(\rho_{S}\right)\right)\right).$$

It is shown in [42] that for a general channel $N_{S\to S^{\prime }}$ the following inequalities hold

(67) $$Q\left(N_{S\to S^{\prime }},a^{†}a,N\right)\leq I_{R}\left(N_{S\to S^{\prime }}\right)\leq Q_{S↔S^{\prime }}^{LOCC}\left(N_{S\to S^{\prime }},a^{†}a,N\right)$$

For the bosonic dephasing channel, we know that the quantum capacity is achieved by using a mixture of Fock states as input, which is invariant under the action of the channel, namely

(68) $$Q\left(N_{S\to S}^{\gamma }\right)=\underset{\rho_{S}^{\prime }}{\sup}\left(S\left(\rho_{S}^{\prime }\right)-S\left(N_{S\to E}^{\gamma^{c}}\left(\rho_{S}^{\prime }\right)\right)\right)$$

where $\rho_{S}^{\prime }$ belongs to the set of mixture of Fock states [20]. In Appendix A, we show that the quantum capacity of the bosonic dephasing channel and its reverse coherent information are equal:

(69) $$I_{R}\left(N_{S\to S}^{\gamma }\right)=Q\left(N_{S\to S}^{\gamma }\right)$$

As constraining the average input energy within a bounded error leads to truncating the Hilbert space dimension and the arguments supporting the equality in (69) are valid over the truncated Hilbert space dimension (see Appendix B), we conclude that for a bosonic dephasing channel, the lower bound on its energy-constrained two-way LOCC-assisted quantum capacity $Q_{S↔S^{\prime }}^{LOCC}\left(N_{S\to S}^{\gamma },a^{†}a,N\right)$ is equal to its energy-constrained quantum capacity with parameter γ. Therefore, taking into account Equations (65), (67) and (69) we arrive at

(70) $$\begin{array}{c} Q\left(N_{S\to S}^{\gamma }\;,a^{†}a,N\right)\leq Q_{S↔S}^{LOCC}\left(N^{\gamma },a^{†}a,N\right)\leq Q\left(N_{S\to S}^{\frac{\gamma }{2}}\;,a^{†}a,N\right). \end{array}$$

**Figure 2:**
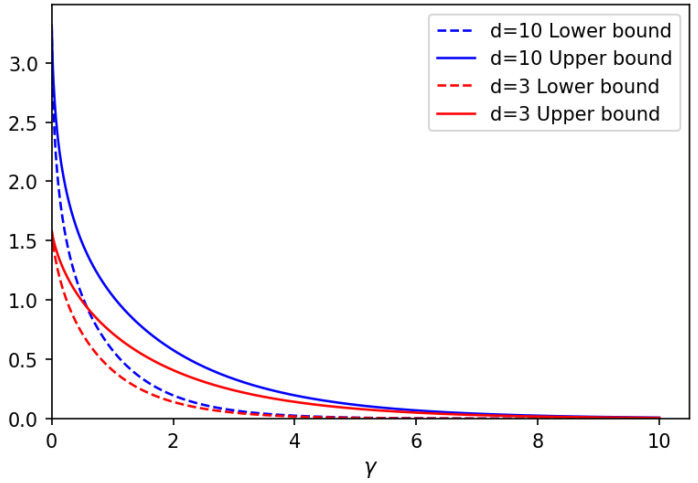
Upper bound and lower bound on energy-constrained LOCC-assisted quantum capacity, $Q_{S↔S}^{LOCC}\left(N^{\gamma },a^{†}a,N\right)$, as given in Equation (70) versus noise parameter γ. Solid lines correspond to the upper bound, while dashed lines correspond to the lower bound. Different colors refer to different dimensions *d* of the truncated Hilbert space and hence to different values of the input energy (N≈d/2).

With reference to [20], we can compute both the lower bound and the upper bound in Equation (70). Figure 2 represents these bounds for Hilbert spaces truncated to the dimension d=3 (red curves), d=10 (blue curves), versus the noise parameter γ. In Figure 2, solid curves correspond to the upper bound in Equation (70), and dashed curves correspond to the lower bound in Equation (70).

**Figure 3:**
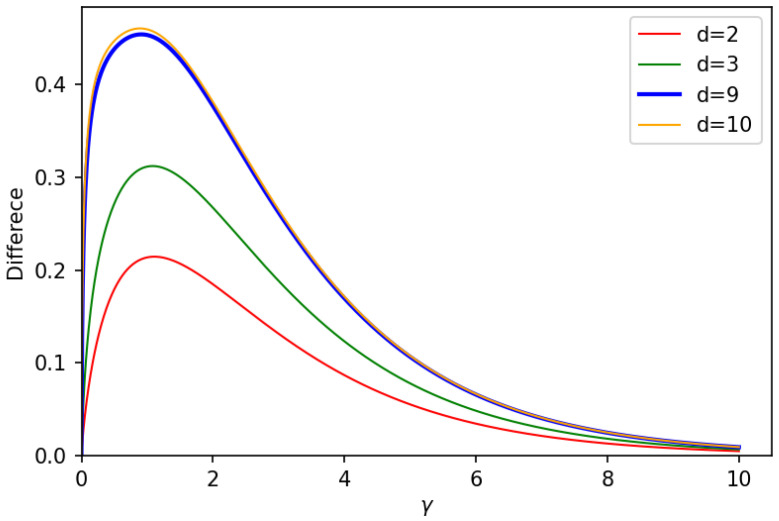
Difference between upper bound and lower bound on $Q_{S↔S}^{LOCC}\left(N^{\gamma },a^{†}a,N\right)$ as given in Equation (70), versus noise parameter γ. Different curves correspond to different dimensions *d* of the truncated Hilbert space and hence to different values of the input energy (N≈d/2).

As one can see in Figure 2, lower bounds and upper bounds are very close to each other, confirming their tightness. To better illustrate this fact, in Figure 3, the difference is shown between the upper and lower bounds versus noise parameter γ for the Hilbert space with dimension d=2 (red curves), d=3 (green curves), d=9 (blue curves), and d=10 (orange curves). As expected, this difference vanishes at γ=0. Furthermore, as it is seen in Figure 3, it decreases as well for large vales of the noise parameter.

**Figure 4:**
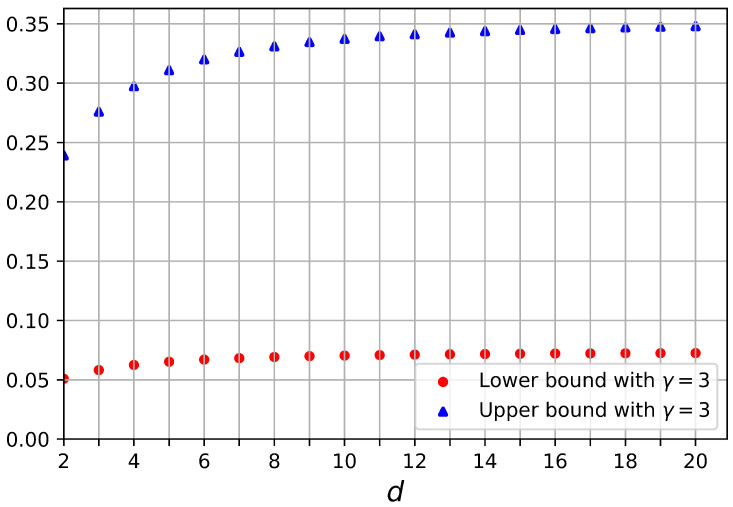
Upper bound (blue triangles) and lower bound (red circles) of $Q_{S↔S}^{LOCC}\left(N_{S\to S}^{\gamma },a^{†}a,N\right)$ given in Equation (70) versus Hilbert space dimension, *d*, when noise parameter γ=3.

As it is shown in Figure 3 for γ<9, the difference between the lower bound and upper bound in Equation (70) increases by increasing the dimension of the Hilbert space. The upper bounds corresponding to different truncated Hilbert spaces are closer to each other for dimensions larger than d=9. The same happens for the lower bounds. The saturation of upper and lower bounds with the increasing Hilbert space dimension can be seen in Figure 4. This effect is in agreement with the results in [20]. There, the quantum capacity of a bosonic dephasing channel was well approximated by the quantum capacity of this channel in a Hilbert space truncated already at dimension nine (capacity saturation effect by increasing input energy in disguise). Therefore, we can conclude that LOCC-assisted quantum capacity of the bosonic dephasing channel without any input energy constraint is upper and lower bounded by the quantum capacity in the following way:

(71) $$Q\left(N_{S\to S}^{\gamma }\right)\leq Q_{S↔S}^{LOCC}\left(N_{S\to S}^{\gamma }\right)\leq Q\left(N_{S\to S}^{\frac{\gamma }{2}}\right).$$

In Figure 4, the lower and upper bounds on the LOCC-assisted quantum capacity of the bosonic dephasing channel do not converge to the same value by increasing *d* (or equivalently *N*); namely, they do not converge to the actual value of the unconstrained capacity (reported in [23]). This is because in the unconstrained case, other more effective approaches, different from the squashing channel, can be employed to obtain tighter bounds [23].

In conclusion, if we use the 50/50 beamsplitter as the squashing channel for a bosonic dephasing channel, we successfully obtain a lower and an upper bound for two-way LOCC-assisted quantum capacity of the bosonic dephasing channel, with energy constraint (Equation (70)) and without energy constraint (Equation (71)). As discussed above, these bounds are tight. For another example of the squashing channel, in the next subsection, we analyze possible candidates among qubit channels.

### 4.2. Qubit Squashing Channels

In this subsection, we truncate the infinite-dimensional Hilbert space into a two-dimensional Hilbert space and search for the best qubit squashing channel. Among the qubit channels, the upper bound for LOCC-assisted quantum capacity of the generalized amplitude damping channel is analyzed by constructing particular squashing channels [46]. Here, our focus is on the bosonic dephasing channel in a truncated two-dimensional Hilbert space, and our approach uses the characterization of symmetric qubit channels [47] to find the one that maximizes mutual information in Equation (55).

Following Equation (52), an optimal input state on the truncated input Hilbert space with dimension two has the following form:

(72) $$\sigma_{SE^{\prime }}^{opt}=\sum_{n=0}^{1}p_{n}{|n\rangle }_{S}\langle n|⊗N_{E\to E^{\prime }}^{sq}\left({|-i\sqrt{\gamma }n\rangle }_{E}\langle -i\sqrt{\gamma }n|\right).$$

Here, $N_{E\to E^{\prime }}^{sq}$ is defined on bounded operators over an infinite-dimensional Hilbert space. However, by truncating the input Hilbert space, the action of squashing channel $N_{E\to E^{\prime }}^{sq}$ is effectively restricted to bounded operators over the Hilbert space spanned by $\left\{|0\rangle ,|-i\sqrt{\gamma }\rangle \right\}$. By employing the Gram–Schmidt procedure, we construct orthonormal states as follows

(73) $$\begin{array}{c} |e_{0}\rangle :=|0\rangle ,\\ |e_{1}\rangle :=\frac{|-i\sqrt{\gamma }\rangle -\langle 0|-i\sqrt{\gamma }\rangle |e_{0}\rangle }{∥\;|-i\sqrt{\gamma }\rangle -\langle 0|-i\sqrt{\gamma }\rangle |e_{0}\rangle ∥\;}. \end{array}$$

Furthermore, as we are restricting our attention to symmetric squashing channels, the input and output spaces of the squashing channel are isomorphic; thus, we denote it by $N_{E\to E}^{sq}$. Hence, the squashing channel in Equation (72) is effectively a qubit channel. Therefore, to derive an upper bound for squashed entanglement of the channel over a two-dimensional Hilbert space, we need to compute the right-hand side of the following inequality from Equation (55)

(74) $$\begin{array}{c} {\tilde{E}}_{sq}\left(N_{S\to S}^{\gamma }\;,a^{†}a,N\right)\leq \underset{p_{n}}{\sup}\left(S\left(\sigma_{S}^{opt}\right)-\underset{N_{E\to E}^{sq}\in N_{sym}}{\sup}I{\left(S;E^{\prime }\right)}_{\sigma_{SE^{\prime }}^{opt}}\right), \end{array}$$

with $\sigma_{SE^{\prime }}^{opt}$ given in Equation (72) and $N_{E\to E}^{sq}$ being a symmetric qubit channels characterized in [47]. These latter are described by either of the following sets of Kraus operators:

(75) $$\begin{array}{c} K_{1}=\left(\begin{array}{cc} \sin(\theta ) & 0\\ 0 & \frac{1}{\sqrt{2}} \end{array}\right)\;\;K_{2}=\left(\begin{array}{cc} 0 & \frac{1}{\sqrt{2}}\\ e^{i\phi }\cos\left(\theta \right) & 0 \end{array}\right). \end{array}$$

and

(76) $$\begin{array}{c} K_{1}^{\prime }=\left(\begin{array}{cc} 1 & 0\\ 0 & \frac{1}{\sqrt{2}}\sin\left(\theta \right) \end{array}\right)\;\;K_{2}^{\prime }=\left(\begin{array}{cc} 0 & \frac{1}{\sqrt{2}}\sin\left(\theta \right)\\ 0 & e^{i\phi }\cos\left(\theta \right) \end{array}\right). \end{array}$$

In both cases, θ∈[0,π] and ϕ∈[0,2π]. In principle, all the terms on the right-hand side of Equation (74) can be computed analytically. However, the final expression after performing the required diagonalization for computing different terms is complicated, and optimization over such expressions is essential. Hence, we perform the optimization on the right-hand side of Equation (74) numerically.

**Figure 5:**
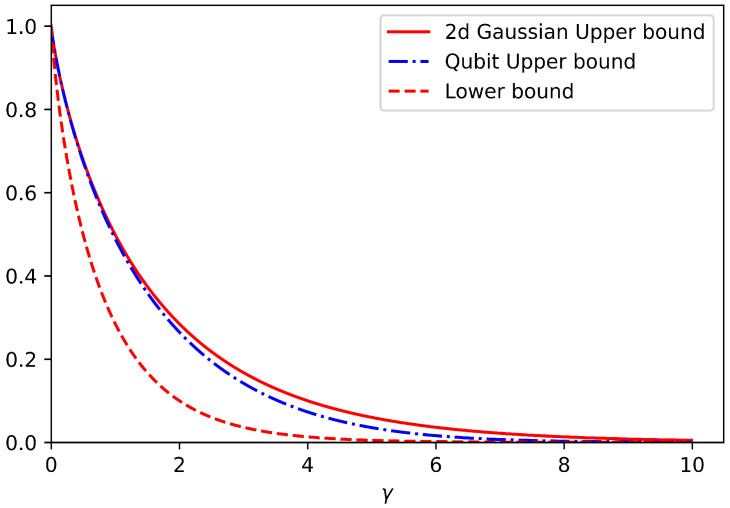
Comparison between upper bounds on energy-constrained LOCC-assisted quantum capacity, $Q_{S↔S}^{LOCC}\left(N^{\gamma },a^{†}a,N\right)$ obtained using one-mode Gaussian symmetric channel (solid red curve) and qubit symmetric channel (dash-dotted blue curve) as functions of the dephasing parameter γ. The red dashed curve shows the lower bound on energy-constrained LOCC-assisted quantum capacity, $Q_{S↔S}^{LOCC}\left(N^{\gamma },a^{†}a,N\right)$ obtained by reverse coherent information.

First, we performed the optimization over all qubit symmetric channels and then found the maximum over all probability distributions. The outcome of our numerical analysis for the upper bound is depicted in Figure 5 with the dashed-dotted blue curve. For better comparison in Figure 5, we also presented the upper bound (solid red line) and the lower bound (dashed red line) when the truncated input Hilbert space is two-dimensional and the squashing channel is a Gaussian channel, as discussed in Section 4.1. Hence, we conclude that, at least for a two-dimensional truncated input Hilbert space, symmetric qubit channels outperform one-mode Gaussian squashing channels for intermediate values of 2<γ<8. However, the difference between the upper bounds given by the 50/50 beamsplitter and symmetric qubit channels is negligible for 2<γ<8 and vanishes for the rest of the values of γ.

## 5. Conclusions

We analyzed the LOCC-assisted quantum capacity of a bosonic dephasing channel subject to energy constraints on input states.

As mentioned earlier, despite the importance of LOCC-assisted quantum capacity, no closed form in terms of entropic quantities exists thus far. Therefore, we focused our attention on computing an upper bound on this quantity using squashed entanglement. However, the existence of nested optimizations (one over the set of input states and the other over the set of isometric extensions of quantum channels) for calculating the squashed entanglement makes it extremely challenging to calculate its value for a given channel. This means that finding an upper bound on the LOCC-assisted quantum capacity is in general a non-trivial and challenging problem.

To overcome these complications for computing the upper bound for LOCC-assisted quantum capacity of a bosonic dephasing channel, first, we used the phase covariant property of the channel to determine the structure of the optimal input state. In other words, we prove that it is sufficient to search for the optimal input state over the set of diagonal states in the Fock basis instead of the whole set of density operators. For the optimization over the isometric extensions of the squashing channels, we were restricted to the set of symmetric channels of the channel environment output. Therein, we chose explicit examples of squashing channels.

First, we considered a 50/50 beamsplitter for the squashing channel. We showed that in this case, the LOCC-assisted quantum capacity of the channel is less than or equal to the quantum capacity of a bosonic dephasing channel, having the noise parameter reduced by a factor two. Furthermore, to derive the tightness possible lower bound, we used the result in [42] where a lower bound on LOCC-assisted quantum capacity is introduced in terms of reverse coherent information. We proved that the reverse coherent information and the quantum capacity of bosonic dephasing channels are equal. Hence, when LOCC is allowed, the reliable rate for sharing entanglement between the two parties increases. Therefore, taking into account the results in [20], we provided computable upper and lower bounds for LOCC-assisted quantum capacity of a bosonic dephasing channel, and we showed that this result is valid whether or not the input state is subject to energy constraints. More importantly, we showed that these bounds are tight, meaning that the quantum capacity of a bosonic dephasing channel with noise parameter γ when LOCC is allowed is very close to the quantum capacity of a bosonic dephasing channel with noise parameter $\frac{\gamma }{2}$. In other words, with the assistance of LOCC, the effective noise parameter is halved.

To extend our analysis beyond a 50/50 beamsplitter squashing channel, we also discussed the case where the squashing channel is a symmetric qubit channel. As put forward in Section 4.2, although the upper bound given by the optimal qubit squashing channel is smaller than the one with the optimal one-mode Gaussian channel for some range of noise parameter, their difference is negligible.

Our results not only set as an explicit example to confirm the importance and tightness of the upper bound in terms of squashed entanglement of the channel, but they also motivate the analysis of the quantum capacity of other non-Gaussian channels, especially with the assistance of LOCC. Additionally, it seems interesting to devote further investigations on characterizing the set of symmetric channels for particular classes of initial states and addressing their performance as squashing channels.

## Data Availability

Data sharing not applicable.

## Appendix A. On the Equality of Quantum Capacity and Reverse Coherent Information for a Bosonic Dephasing Channel

As defined in Equation (66), the reverse coherent information of the bosonic dephasing channel is given by

(A1) $$I_{R}\left(N_{S\to S}^{\gamma }\right)=\underset{\rho_{S}}{\sup}\left(S\left(\rho_{S}\right)-S\left(N_{S\to E}^{\gamma^{c}}\left(\rho_{S}\right)\right)\right).$$

Moreover, its quantum capacity is proven to be given by the following optimization problem where the supremum is taken over a mixture of Fock states $\rho_{S}^{\prime }$ [20]:

(A2) $$Q\left(N_{S\to S}^{\gamma }\right)=\underset{\rho_{S}^{\prime }}{\sup}\left(S\left(\rho_{S}^{\prime }\right)-S\left(N_{S\to E}^{\gamma^{c}}\left(\rho_{S}^{\prime }\right)\right)\right).$$

Here, we show that the supremum in Equation (A1), as in the case of quantum capacity, is achieved by using a mixture of Fock states. Therefore, we prove that

(A3) $$I_{R}\left(N_{S\to S}^{\gamma }\right)=Q\left(N_{S\to S}^{\gamma }\right).$$

To this end, we first define the following function:

(A4) $$I_{R}\left(N_{S\to S},\rho_{S}\right)S\left(\rho_{S}\right)-S\left(N_{S\to E}^{c}\left(\rho_{S}\right)\right).$$

Next, we prove that $I_{R}\left(N_{S\to S},\rho_{S}\right)$ is concave with respect to input states $\rho_{S}$. Consider the following two classical quantum states:

(A5) $$\begin{array}{cc} \sigma_{XS} & =\sum_{n}p_{n}{|n\rangle }_{X}\langle n|⊗\rho_{S}^{\left(n\right)},\\ \tau_{XE} & =\sum_{n}p_{n}{|n\rangle }_{X}\langle n|⊗N_{S\to E}^{c}\left(\rho_{S}^{\left(n\right)}\right), \end{array}$$

where $N_{S\to E}^{c}$ is the complementary channel to the channel $N_{S\to S}$. The following inequalities hold true:

(A6) $$\begin{array}{cc} I{\left(X;E\right)}_{\tau_{XE}} & \leq I{\left(X;S\right)}_{\sigma_{XS}},\\ S\left(\tau_{E}\right)-S{\left(E|X\right)}_{\tau_{XE}} & \leq S\left(\sigma_{S}\right)-S{\left(S|X\right)}_{\sigma_{XS}},\\ S{\left(S|X\right)}_{\sigma_{XS}}-S{\left(E|X\right)}_{\tau_{XE}} & \leq S\left(\sigma_{S}\right)-S\left(\tau_{E}\right). \end{array}$$

The first inequality is due to the data-processing inequality of mutual information, the second inequality follows the definition of mutual information, and the third inequality is just a rearrangement. Therefore, due to the classical quantum nature of the states $\sigma_{XS},\tau_{XE}$ in Equation (A5), and the last inequality of (A6), we have:

(A7) $$\begin{array}{c} \sum_{n}p_{n}\left(S\left(\rho_{S}^{\left(n\right)}\right)-S\left(N_{S\to E}^{c}\left(\rho_{S}^{\left(n\right)}\right)\right)\right)\leq S\left(\sum_{n}p_{n}\rho_{S}^{\left(n\right)}\right)-S\left(\sum_{n}p_{n}N_{S\to E}^{c}\left(\rho_{S}^{\left(n\right)}\right)\right), \end{array}$$

which is equivalent to

(A8) $$\sum_{n}p_{n}I_{R}\left(N_{S\to S},\rho_{S}^{\left(n\right)}\right)\leq I_{R}\left(N_{S\to S},\sum_{n}p_{n}\rho_{S}^{\left(n\right)}\right),$$

for any probability distribution $p_{n}$. As a consequence, $I_{R}\left(N_{S\to S},\rho_{S}\right)$ is a concave function with respect to its argument $\rho_{S}$.

On the other hand, according to Equation (30), the complementary channel of a bosonic dephasing channel is invariant under the phase shift operator of Equation (28). Then, by considering the unitarily invariance property of the von Neumann entropy along with the invariance property of the complementary channel of the bosonic dephasing channel under the action of $U_{\theta }$, we conclude that:

(A9) $$I_{R}\left(N_{S\to S}^{\gamma },\rho_{S}\left(\theta \right)\right)=I_{R}\left(N_{S\to S}^{\gamma },\rho_{S}\right)$$

where $\rho_{S}(\theta ):=U_{\theta }\rho_{S}U_{\theta }$. Employing the concavity of $I_{R}\left(N_{S\to S^{\prime }},\rho_{S}\right)$ as in Equation (A8) and Equation (A9), we are led to:

(A10) $$I_{R}\left(N_{S\to S}^{\gamma },\rho_{S}\right)\leq I_{R}\left(N_{S\to S}^{\gamma },\int_{0}^{2\pi }d\theta p\left(\theta \right)\rho_{S}\left(\theta \right)\right).$$

Taking p(θ) as a flat distribution and expanding $\rho_{S}$ in the Fock basis, $\rho_{S}=\sum_{m,n}\rho_{m,n}{|m\rangle }_{S}\langle n|$, we have:

(A11) $$\int_{0}^{2\pi }\rho_{S}\left(\theta \right)p\left(\theta \right)d\theta =\frac{1}{2\pi }\sum_{m,n}\int_{0}^{2\pi }d\theta \rho_{m,n}e^{i\theta (n-m)}{|n\rangle }_{S}\langle m|.$$

Inserting the above result into the right-hand side of (A10), we end up with:

(A12) $$I_{R}\left(N_{S\to S}^{\gamma },\rho_{S}\right)\leq I_{R}\left(N_{S\to S}^{\gamma },\sum_{n}\rho_{n,n}{|n\rangle }_{S}\langle n|\right).$$

Hence, the supremum in Equation (A1) is achieved by a mixture of Fock states $\rho_{S}^{\prime }$. In other words, the optimization in Equations (A1) and (A2) are over the same space, and this proves their equality as expressed in Equation (A3).

## Appendix B. Bounding the Errors Due to Space Trucation

Since Equation (69) involves entropic quantities, we show here how to bound the error on the von Neumann entropy S(ρ) when constraining the average input energy $\mathrm{Tr}(a^{†}a\rho )$ within an error ϵ.

Since, according to Section 3, the optimal input state is diagonal in the Fock basis, let us just consider $\rho =\sum_{n=0}^{\infty }p_{n}\left|n\rangle \langle n\right|$, with $\mathrm{Tr}\left(a^{†}a\rho \right)=\sum_{n=0}^{\infty }np_{n}=N$.

Now, for a given ϵ>0, suppose we truncate the space at $dim\;H=d_{\epsilon }$ such that

(A13) $$\sum_{n=0}^{d_{\epsilon }}np_{n}=N-\epsilon ,$$

where *N* is the average input energy. Then, consider the entropy S(ρ). It is $S\left(\rho \right)=H({\left\{p_{n}\right\}}_{n=0}^{\infty })$. Therefore,

(A14) $$H\left({\left\{p_{n}\right\}}_{n=0}^{\infty }\right)=\sum_{n=0}^{d_{\epsilon }}p_{n}\left(-\log p_{n}\right)+\sum_{n=d_{\epsilon }+1}^{\infty }p_{n}\left(-\log p_{n}\right).$$

It follows that

(A15) $$\sum_{n=d_{\epsilon }+1}^{\infty }p_{n}\left(-\log p_{n}\right)\leq H\left({\left\{p_{n}\right\}}_{n=0}^{d_{\epsilon }}\right)\leq H\left({\left\{{\tilde{p}}_{n}\right\}}_{n=0}^{d_{\epsilon }}\right),$$

where $H\left({\left\{p_{n}\right\}}_{n=0}^{d_{\epsilon }}\right)=\sum_{n=0}^{d_{\epsilon }}p_{n}\left(-\log p_{n}\right)$ and $H\left({\left\{{\tilde{p}}_{n}\right\}}_{n=0}^{d_{\epsilon }}\right)=\sum_{n=0}^{d_{\epsilon }}{\tilde{p}}_{n}\left(-\log{\tilde{p}}_{n}\right)$ with ${\left\{{\tilde{p}}_{n}\right\}}_{n=0}^{d_{\epsilon }}$ are the optimal distribution satisfying $\sum_{n=0}^{d_{\epsilon }}n{\tilde{p}}_{n}=N-\epsilon$.

The first inequality in (A15) is guaranteed by the fact that for $d_{\epsilon }$ that is sufficiently large, the first term is the reminder of a converging series, while the second term is the partial sum.

Since ${\left\{p_{n}\right\}}_{n=0}^{d_{\epsilon }}$ satisfies (A13), the second inequality in (A15) follows from the fact that ${\left\{{\tilde{p}}_{n}\right\}}_{n=0}^{d_{\epsilon }}$ maximizes $H({\left\{p_{n}\right\}}_{n=0}^{d_{\epsilon }})$.

Finally, the farthest right term in (A15) depends on ϵ; hence, it provides a bound on the error $\sum_{n=d_{\epsilon }+1}^{\infty }p_{n}\left(-\log p_{n}\right)$ of S(ρ) when we truncate the space to $dim\;H=d_{\epsilon }$.
